# Comparison of Endoscopic Ultrasound-Guided Fine-Needle Aspiration and Biopsy Device for Lymphadenopathy

**DOI:** 10.1155/2021/6640862

**Published:** 2021-04-15

**Authors:** Yuki Tanisaka, Masafumi Mizuide, Akashi Fujita, Tomoya Ogawa, Ryuichiro Araki, Masahiro Suzuki, Hiromune Katsuda, Youichi Saito, Kazuya Miyaguchi, Tomoaki Tashima, Yumi Mashimo, Masami Yasuda, Shomei Ryozawa

**Affiliations:** ^1^Department of Gastroenterology, Saitama Medical University International Medical Center, Japan; ^2^Saitama Medical University, Community Health Science Center, Japan; ^3^Department of Pathology, Saitama Medical University International Medical Center, Japan

## Abstract

**Background:**

Accurate diagnosis of benign and malignant lymphadenopathy is important for determining the appropriate treatment and prognosis. This study evaluated the diagnostic accuracy and usefulness of endoscopic ultrasound-guided fine-needle aspiration (EUS-FNA) with a conventional needle compared to endoscopic ultrasound-guided fine-needle biopsy (EUS-FNB) with a Franseen needle for diagnosing lymphadenopathy.

**Methods:**

Patients who underwent EUS-FNA or EUS-FNB for mediastinal or abdominal lymphadenopathy between July 2013 and August 2020 were enrolled in the study. The outcomes between EUS-FNA patients (July 2013 to January 2017; 22-gauge conventional needle; Group A) and EUS-FNB patients (February 2017 to August 2020; 22-gauge Franseen needle; Group B) were compared.

**Results:**

A total of 154 patients (Group A: 83; Group B: 71) were analyzed. The diagnostic accuracy (differentiating between malignant and benign lesions) was 88.0% (95% confidence interval [CI], 79.2–93.3%) in Group A and 95.8% (95% CI, 88.3–98.8%) in Group B. Group B had high diagnostic accuracy, but there was no difference between the groups (*p* = 0.14). Group B had significantly fewer passes (median 2, interquartile range (IQR): 2-4) than Group A (median 3, IQR: 3-4) (*p* < 0.001). No procedural adverse events occurred in either group.

**Conclusions:**

Although the diagnostic accuracy between the groups was not statistically significant, EUS-FNB with a Franseen needle provided high diagnostic accuracy and required fewer passes to establish a diagnosis. Thus, EUS-FNB is useful for diagnosing lymphadenopathy.

## 1. Introduction

Mediastinal and abdominal lymphadenopathy present with numerous symptoms, and accurate diagnosis is important to determine the appropriate treatment and prognosis [1]. Although cross-sectional imaging, such as computed tomography, magnetic resonance imaging, and positron emission tomography, is useful for detecting lymphadenopathy, it is difficult to distinguish between benign and malignant lesions using only imaging modalities [2–4]. Invasive procedures, such as open thoracic surgery, thoracoscopy, and laparoscopy, were previously required for histological diagnosis.

Endoscopic ultrasound (EUS) can easily access the lymph nodes and provide detailed information on the shape, diameter, and internal echoic features via high-resolution images [5–7]. The features of malignant lymph nodes reported on EUS images are a diameter of 10 mm or greater, round shape, sharply demarcated borders, and a homogeneous and hypoechoic central echo pattern. EUS-guided fine-needle aspiration (EUS-FNA) has been used for the diagnosis of many lesions since it was first reported in 1992 [8]. EUS-FNA is a minimally invasive method for collecting diagnostic cytological and histological materials for lymphadenopathy compared to surgery. Moreover, it can be used to determine cancer stages and the etiology and surgically resect malignancy recurrences. Therefore, EUS-FNA is useful for the lymphadenopathy diagnostic process [9–16]. A recent systematic review and meta-analysis reported that the pooled sensitivity and specificity were 87% and 100%, for differentiating benign and malignant lymphadenopathy, respectively [17]. However, fine-needle aspiration (FNA) tissue sampling sometimes cannot provide enough material for diagnosis.

With the recent progress of needles, the fine-needle biopsy (FNB) device, designed primarily to obtain core tissue samples, was introduced to overcome the FNA sampling material limitation [18, 19]. The usefulness of the FNB needle has been extensively reported [20, 21]. Immunostaining is often required for lymphadenopathy diagnosis and requires a large tissue sample, which can be provided by the FNB needle. The Franseen needle, a type of FNB needle used in our facility, has a crown tip with three symmetrical surfaces that manifest as three cutting edges. This unique design is expected to obtain adequate tissue amounts. Moreover, the needle is made of cobalt-chromium, a highly durable alloy; this allows repeat punctures without needle dysfunction. Previous reports have shown that large tissue sample amounts were obtained using a Franseen needle [19, 22].

Previous studies have compared the utility of conventional and FNB needles for diagnosing lymphadenopathy in a small number of cases [23–25]. Therefore, this study was aimed to compare the diagnostic accuracy and usefulness between EUS-FNA with a conventional needle and EUS-FNB with a Franseen needle for diagnosing lymphadenopathy.

## 2. Methods

This is a retrospective, single-center study approved by the Institutional Review Board at Saitama Medical University International Medical Center (18-253). All patients provided written informed consent before undergoing the procedure.

### 2.1. Patients

We planned to perform EUS-FNA or EUS-FNB in patients with mediastinal or abdominal lymphadenopathy that could not be diagnosed using only cross-sectional imaging at our facility between July 2013 and August 2020. The inclusion criteria were as follows: lymphadenopathy >10 mm in maximal diameter; and lymphadenopathy indicated using cross-sectional imaging to be approachable with EUS-FNA or EUS-FNB from the esophagus, stomach, or duodenum. The exclusion criteria were as follows: superficial lymphadenopathy that could permit a superficial approach to obtain tissue; patients treated with antithrombotic agents that may not be discontinued; lymphadenopathy performed via EUS-FNA or EUS-FNB using a 25-gauge needle (Expect®, Boston Scientific, Marlborough, MA, USA). Patients were divided into two groups: Group A for EUS-FNA (July 2013 to January 2017; 22-gauge conventional needle) and Group B for EUS-FNB (February 2017 to August 2020; 22-gauge Franseen needle).

### 2.2. Procedures

EUS procedures were performed by four training fellows with experience in less than 50 EUS-FNA procedures under the direct supervision of endoscopists with experience in over 100 EUS-FNA procedures. In cases where the training fellows encountered difficulties in completing the procedure, the experienced endoscopists completed it. Two training fellows performed procedures in each group. They did not perform procedures across both groups. All procedures were performed with the patient under conscious sedation via a combination of intravenous midazolam and pethidine.

The EUS-FNA and EUS-FNB procedures used a linear echoendoscope (GF-UCT260; Olympus Medical Systems, Tokyo, Japan) paired with an ultrasound system (EU-ME2 Premier Plus; Olympus Medical Systems, Tokyo, Japan). The swollen lymph node was visualized, the regional vasculature was excluded using the color Doppler mode, and then, the target lesion was punctured. Next, the stylet was removed, and continuous suction was applied using a 20 mL syringe. After 20 rapid suction strokes within the lesion, the suction was released, and the needle was removed. The obtained tissue specimens were pushed out by reinserting the stylet or creating positive pressure using air.

Rapid on-site evaluation (ROSE) is not available at our facility. Thus, the sample was examined macroscopically to determine whether the specimen was of a sufficient quantity. We also checked the color of specimens, because a red specimen was thought to be blood while a white specimen was thought to be tissue. These examinations were performed under the supervision of a pathology technician. When a certain amount of specimen (including a certain proportion of white specimen) was obtained, we finished the procedure. If the macroscopic results showed an increase in blood components, the negative pressure level of the syringe was decreased or no suction was applied, as necessary.

**Figure 1:**
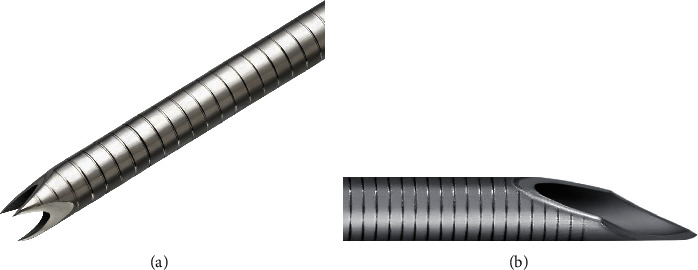
Endoscopic ultrasound-guided fine needles. (a) A Franseen needle with three symmetric cutting tips for endoscopic ultrasound-guided fine-needle biopsy (EUS-FNB). (b) A conventional needle for endoscopic ultrasound-guided fine-needle aspiration (EUS-FNA).

The Expect® conventional needle (Boston Scientific, Marlborough, MA, USA) was used for Group A, and the Acquire® Franseen needle (Boston Scientific, Marlborough, MA, USA) was used for Group B (Figure 1).

### 2.3. Sampling Evaluation

Smears were alcohol-fixed (95% ethanol, room temperature for 15 min), and subsequently, Papanicolau stained in the laboratory for cytological examination. The sample was checked for adequacy and then preserved in 10% neutral buffered formalin, embedded in paraffin, and cut into 4 *μ*m thick serial sections for hematoxylin and eosin staining for histological examination. Immunostaining was performed if needed. Two pathology technicians and two pathologists examined the sections.

### 2.4. Definitions and Outcome Measurement

Patients' age, sex, lymph node diameter, lymph node location, puncture site, number of passes, procedure time, and final diagnosis were recorded from electronic medical records. The final diagnosis was classified as malignant or benign lymph nodes, and the sensitivity, specificity, positive predictive value, negative predictive value, and accuracy for differentiating between malignant and benign lesions were estimated. The final diagnosis was confirmed through histological evaluation of the resected specimen when surgery was performed. For patients who did not undergo surgery, the final diagnosis was defined as malignant when histological evaluation of EUS-FNA- or EUS-FNB-based tissue confirmed malignancy and the lymph node became larger on follow-up imaging evaluations. In patients without histological confirmation, the final diagnosis was defined as malignant when the clinical course or cross-sectional imaging evaluations consistently worsened during the follow-up period. The final diagnosis was defined as benign if malignant findings were absent on histological examinations and there was no progression after more than six months of follow-up. Technical success was defined as EUS-FNA or EUS-FNB completion with a successful acquisition of macroscopically visible whitish material.

### 2.5. Statistical Analysis

The Shapiro-Wilk's W test showed that all continuous variables were not normally distributed. Thereby, these variables were expressed as medians and interquartile ranges (IQR), and the Mann–Whitney *U* test was used to compare continuous data. Between-group categorical data comparisons were performed using Fisher's exact test to calculate 2-tailed *p* values; *p* < 0.05 was considered statistically significant. Statistical data were calculated using SAS JMP (version 14.2.0; SAS Institute Inc., Cary, NC, USA).

## 3. Results

### 3.1. Patients

**Figure 2:**
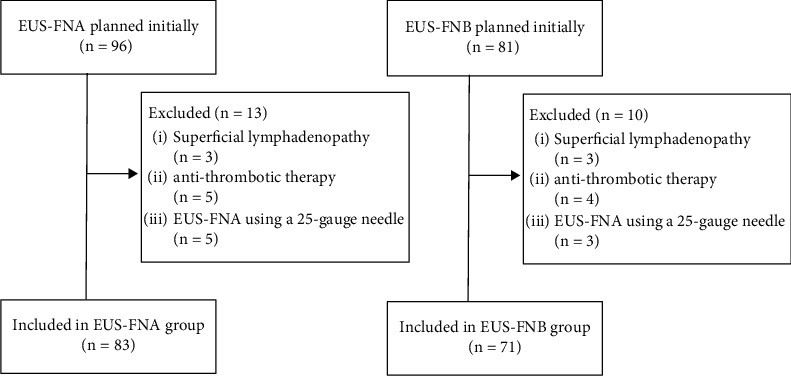
A flowchart of patients' selection between EUS-FNA and EUS-FNB groups.

Figure 2 provides a flowchart of patients' selection. In the EUS-FNA group (July 2013 to January 2017), 96 patients were initially included. We excluded 3 patients with superficial lymphadenopathy, 5 patients with antithrombotic therapy, and 5 patients who underwent EUS-FNA using a 25-gauge needle. Finally, 83 patients were included in the EUS-FNA group (Group A).

In the EUS-FNB group (February 2017 to August 2020), 81 patients were initially included. We excluded 3 patients with superficial lymphadenopathy, 4 patients with antithrombotic therapy, and 3 patients who underwent EUS-FNB using a 25-gauge needle. Finally, 71 patients were included in the EUS-FNB group (Group B). A total of 154 patients (Group A: 83; Group B: 71) were analyzed. The patient characteristics of Group A and Group B are presented in Table 1. Age, sex, lymph node diameter, and lymph node location did not significantly differ between the groups. A 25-gauge needle was used when the lymphadenopathy was small (within 15 mm) and located near the vessel.

### 3.2. Final Diagnoses

The final diagnoses are presented in Table 2. A total of 138 patients had malignant lesions (metastatic, 88 (Group A: 42, Group B: 46); malignant lymphoma, 50 (Group A: 33, Group B: 17)), and 16 had benign lesions (reactive change, 15 (Group A: 8, Group B: 7); sarcoidosis, 1 (Group A: 1)). The diagnosis that was obtained through surgery was in 3 cases. The lymphadenopathy was located in the para-aortic region in one case. Although lymphoma was the chief probable diagnosis, the results of EUS-FNA showed no malignancy. Finally, open surgical resection from the lymphadenopathy was performed. The final diagnosis was T-cell lymphoma. In 2 cases, the lymphadenopathy was located near pancreatic cancer and duodenal cancer lesions. It was difficult to obtain sufficient samples from the main lesions to make a correct diagnosis. Therefore, EUS-FNB was performed in the lymphadenopathy near the main lesions. Finally, the main lesions were resected, including the lymphadenopathy, and they were diagnosed as adenocarcinoma. However, we must be mindful that it is difficult to confirm whether the lymph node resected and evaluated to establish the final diagnosis was the one we sampled during EUS.

We evaluated EUS findings [5–7]. As the diameter was 10 mm or greater in all cases, we evaluated whether sharply demarcated borders, round shape, and homogeneous and hypoechoic central echo pattern were present. Sharply demarcated borders, round shape, and homogeneous and hypoechoic central echo pattern were found in 45.7%, 49.3%, and 61.6% of malignant lesions and in 37.5%, 18.8%, and 56.3% of benign lesions (Table 3), respectively. Although assessment of EUS findings was useful, it was difficult to reach a correct diagnosis without EUS-FNA or EUS-FNB.

### 3.3. Procedure Outcomes between Conventional and Franseen Needle Groups

Procedural outcome comparisons between the conventional (Group A) and Franseen (Group B) needle groups are presented in Table 4. Group B had significantly fewer passes (median 2, IQR: 2-4) than Group A (median 3, IQR: 3-4) (*p* < 0.001). There were no differences in procedure time, and the technical success rate was 100% in both groups. No procedural adverse events occurred in either group. We also performed a subgroup analysis regarding malignant lymphoma. Group B had significantly fewer passes (median 3, IQR: 3-4) than Group A (median 4, IQR: 4-5) (*p* < 0.001). There were no differences in procedure time (Table 5).

### 3.4. Histological Diagnostic Accuracy between Conventional and Franseen Needle Groups

Histological diagnostic accuracy comparisons between the conventional (Group A) and Franseen (Group B) needle groups are presented in Table 6. Diagnostic accuracy did not differ according to the puncture route, tumor type, and tumor size. The diagnostic accuracy from transduodenal puncture was higher in Group B (92.3%) than in Group A (69.2%), but this difference was not statistically significant (*p* = 0.15). Moreover, the diagnostic accuracy of metastasis in Group B (93.5%) was higher than that in Group A (81.0%), but this difference was also not statistically significant (*p* = 0.11). We also performed a subgroup analysis regarding malignant lymphoma. Diagnostic accuracy did not differ according to the puncture route and tumor size (Table 5).

### 3.5. Diagnostic Performance for Differentiating Malignant and Benign Lesions

Histological diagnostic performance comparisons for differentiating between malignant and benign lesions are presented in Table 7. The results were as follows: sensitivity (Group A: 86.7% [65/75]; Group B: 95.2% [60/63]), specificity (Group A: 100% [8/8]; Group B: 100% [8/8]), positive predictive value (Group A: 100% [65/65]; Group B: 100% [60/60]), negative predictive value (Group A: 44.4% [8/18]; Group B: 72.7% [8/11]), and accuracy (Group A: 88.0% [73/83], 95% confidence interval [CI], 79.2–93.3%; Group B: 95.8% [68/71], 95% CI, 88.3–98.8%). Group B had a higher diagnostic accuracy, but there was not significantly different (*p* = 0.14). We also analyzed the diagnostic accuracy using cytology. It was 83.1% (95% CI, 73.7–89.7%) for EUS-FNA (Group A) and 91.5% (95% CI, 82.8–96.1%) for EUS-FNB (Group B). Group B had high diagnostic accuracy, but there was no difference between the groups (*p* = 0.15). Moreover, combined diagnostic accuracy using cytology and histology was 90.4% (95% CI, 82.1–95.0%) in Group A and 95.8% (95% CI, 88.3–98.8%) in Group B. There was no difference between the groups (*p* = 0.23) (Table 8).

## 4. Discussion

Previous reports compared the utility of conventional and FNB needles for diagnosing lymphadenopathy in only a small number of cases [23–25]. Thus, we conducted a comparison study using a larger number. In this study, the diagnostic accuracy for differentiating between malignant and benign lymphadenopathy using a Franseen needle (Group B) was higher than that of the accuracy using a conventional needle (Group A) (95.8% vs. 88%), although this difference was not statistically significant. Previous reports stated that the lymphadenopathy diagnostic accuracy using a conventional needle was over 85% [9–16], making it difficult to show significant differences compared to that obtained using a Franseen needle.

In this study, the Franseen needle showed several benefits. First, the Franseen needle required significantly fewer passes than the conventional needle with no available ROSE, even in cases of malignant lymphoma. Although we did not evaluate the accurate amount of tissue, this result may suggest that larger amounts of tissue could be acquired using a Franseen needle, making it easier to perform the macroscopic identification of tissue samples. Although ROSE improves diagnostic accuracy [26], it is not always available, even in high-volume centers such as ours. In such facilities, the FNB needle facilitates the macroscopic examination of tissue samples. Moreover, fewer passes minimize the risk of trauma to the tissue or vessels. Second, the diagnostic accuracy of metastasis using a Franseen needle was high (although not significantly different between the groups). For indeterminate lymphadenopathy diagnosis (i.e., the cancer etiology cannot be identified by cross-sectional imaging or EUS findings), larger amounts of tissue are useful for the correct diagnosis. More tissue is also beneficial for immunostaining. If the EUS-FNB and surgical specimens are histologically similar, recurrent cancer is diagnosed. Thus, a larger specimen is more useful for cancer diagnosis, and the Franseen needle could provide this. Third, the EUS-FNB technical success rate was 100% despite the hypothesis that the Franseen needle tip may make puncturing difficult. This was particularly worrisome for transduodenal punctures, but the accurate diagnosis of transduodenal punctures was extremely satisfactory (92.3%). The Franseen needle tip may induce more localized trauma, resulting in more frequent adverse events such as bleeding. Fortunately, there was no adverse event using a Franseen needle in this study. However, we must be mindful that adverse events, such as bleeding, can occur with a Franseen needle.

This study is retrospective and was conducted over two distinct periods; EUS-FNA was performed in the first period (in 83 patients) and EUS-FNB in the second (in 71 patients). Therefore, the learning curve must be taken into consideration. All procedures were initially performed by training fellows with less experience under the direct supervision of experienced endoscopists, and training fellows did not perform procedures across both groups. Therefore, we investigated the diagnostic accuracy differences between the beginning and end of each period using EUS-FNA (beginning: 42 cases, end: 41 cases) and EUS-FNB (beginning: 36 cases, end: 35 cases) to determine the influence of the learning curve. The diagnostic accuracies of the EUS-FNA in the beginning and end periods were 90.5% (38/42) and 85.4% (35/41), respectively, and there was no difference between the periods (*p* = 0.52). The diagnostic accuracies of the EUS-FNB in the beginning and end periods were 97.2% (35/36) and 94.3% (33/35), respectively, also with no difference between the periods (*p* = 0.61). The learning curve cannot be completely discounted, but it does not have a considerable influence on outcomes.

EUS-FNB allows for a wide range of tests for personalized diagnosis. Moreover, it enables genetic testing, which is useful in the individualized medicine era [27]. Hence, EUS-FNB may expand the available diagnosis and treatment tools, even for lymphadenopathy.

The fork-tip needle is another EUS-FNB needle which provides a high rate of histological specimens [28], although we have not used it in this study. This needle is also useful for the diagnosis of lymphadenopathy.

This study has several limitations. First, it was a single-center retrospective analysis. Moreover, it was not a side-by-side comparative study, and not randomized. Second, as previously mentioned, the procedure was performed by four endosonographers-in-training, which carries a heterogeneity risk between operators despite having been performed under the direct supervision of experienced endoscopists. A prospective study with a larger number of cases is necessary. Moreover, we used only the continuous suction technique using a 20 mL syringe. Continuous suction with the stylet technique (the stylet slow-pull technique), which has been reported as useful to avoid blood contamination [29–31], would be useful for lymphadenopathy in future studies.

In conclusion, although there was no difference in the diagnostic accuracy between the groups, EUS-FNB with a Franseen needle provided high diagnostic accuracy and required fewer passes to establish a diagnosis. Therefore, EUS-FNB is useful for diagnosing lymphadenopathy.

## Figures and Tables

**Table 1:** Patient characteristics between conventional (Group A) and Franseen (Group B) needle groups.

	Group A	Group B	*p* value
Patients, *n* (%)	83	71	
Age, median (IQR), years	67 (60-74)	70 (61-74)	0.38
Gender (male), *n* (%)	51 (61.4)	36 (50.7)	0.20
Diameter of lymph node, median (IQR), mm	22 (15-35)	26 (19-35)	0.32
Location of lymph node, *n* (%)			>0.99
Abdominal lymphadenopathy	77 (92.8)	65 (91.5)	
Mediastinum lymphadenopathy	6 (7.2)	6 (8.5)	

*n*: number of cases; IQR: interquartile range.

**Table 2:** Final lymphadenopathy diagnoses (*n* = 154).

Final diagnosis	*n* (%)
Total of malignant lesions	138 (89.6) (Group A: 75, Group B: 63)
Metastasis	88 (57.1) (Group A: 42, Group B: 46)
Malignant lymphoma	50 (32.5) (Group A: 33, Group B: 17)
Total of benign lesions	16 (10.4) (Group A: 8, Group B: 8)
Reactive change	15 (9.7) (Group A: 8, Group B: 7)
Sarcoidosis	1 (0.7) (Group A: 1)

Group A: conventional needle group Group B: Franseen needle group. *n*: number of cases.

**Table 3:** EUS findings on malignant and benign lymphadenopathy.

	Sharply demarcated border	Rounded shape	Homogeneous and hypoechoic central echo pattern
Malignant lesions, *n* (%)	63/138 (45.7)	68/138 (49.3)	85/138 (61.6)
Benign lesions, *n* (%)	6/16 (37.5)	3/16 (18.8)	9/16 (56.3)

*n*: number of cases.

**Table 4:** Procedural comparisons between the conventional (Group A) and Franseen (Group B) needle groups.

	Group A	Group B	*p* value
Number of punctures, median (IQR)	3 (3-4)	2 (2-4)	<0.001
Procedure time (min), median (IQR)	27 (20-32)	26 (23-29)	0.32
Technical success, *n* (%)	83/83 (100)	71/71 (100)	>0.99
Adverse events, *n* (%)	0/83 (0)	0/71 (0)	>0.99

*n*: number of cases; IQR: interquartile range.

**Table 5:** Comparison of procedure results and diagnostic accuracy between conventional (Group A) needle and Franseen (Group B) needle groups in malignant lymphoma.

	Group A	Group B	*p* value
Number of punctures, median (IQR)	4 (4-5)	3 (3-4)	<0.001
Procedure time (min), median (IQR)	27 (22-31)	27 (24-30)	0.92
Diagnostic accuracy, *n* (%)			
Puncture route			
Transesophageal	1/1 (100)	0/0	>0.99
Transgastric	25/27 (92.6)	10/10 (100)	>0.99
Transduodenal	5/5 (100)	7/7 (100)	>0.99
Tumor size			
≥20 mm	23/24 (95.8)	15/15 (100)	>0.99
<20 mm	8/9 (88.9)	2/2 (100)	>0.99
Overall	31/33 (94.0)	17/17 (100)	0.54

*n*: number of cases; IQR: interquartile range.

**Table 6:** Histological diagnostic accuracy comparisons between the conventional (Group A) and Franseen (Group B) needle groups.

Diagnostic accuracy, *n* (%)	Group A	Group B	*p* value
Puncture route			
Transesophageal	5/6 (83.3)	6/6 (100)	>0.99
Transgastric	59/64 (92.2)	38/39 (97.4)	0.40
Transduodenal	9/13 (69.2)	24/26 (92.3)	0.15
Tumor type			
Metastasis	34/42 (81.0)	43/46 (93.5)	0.11
Malignant lymphoma	31/33 (94.0)	17/17 (100)	0.54
Benign lesions	8/8 (100)	8/8 (100)	>0.99
Tumor size			
≥20 mm	49/55 (89.1)	44/46 (95.7)	0.29
<20 mm	24/28 (85.7)	24/25 (96)	0.35
Overall	73/83 (88.0)	68/71 (95.8)	0.14

*n*: number of cases.

**Table 7:** Histological diagnostic performance comparisons for differentiating between malignant and benign lesions.

Diagnostic accuracy, *n* (%), 95% CI	Group A	Group B	*p* value
Sensitivity	65/75 (86.7)	60/63 (95.2)	0.14
	77.2-92.6	87.0-98.7	
Specificity	8/8 (100)	8/8 (100)	>0.99
	67.6-100	67.6-100	
Positive predictive value	65/65 (100)	60/60 (100)	>0.99
	94.4-100	94.0-100	
Negative predictive value	8/18 (44.4)	8/11 (72.7)	0.25
	24.6-66.3	43.4-90.3	
Accuracy	73/83 (88.0)	68/71 (95.8)	0.14
	79.2-93.3	88.3-98.8	

*n*: number of cases; CI: confidence interval.

**Table 8:** Diagnostic accuracy comparisons between the conventional (Group A) and Franseen (Group B) needle groups regarding cytology, histology, and combined cytology and histology.

Diagnostic accuracy, *n* (%), 95% CI	Group A	Group B	*p* value
Cytology	69/83 (83.1)	65/71 (91.5)	0.15
	73.7–89.7	82.8–96.1	
Histology	73/83 (88.0)	68/71 (95.8)	0.14
	79.2-93.3	88.3-98.8	
Combined cytology and histology	75/83 (90.4)	68/71 (95.8)	0.23
	82.1–95.0	88.3-98.8	

*n*: number of cases; CI: confidence interval.

## Data Availability

The patient data used to support the findings of this study are restricted by the Institutional Review Board at Saitama Medical University International Medical Center in order to protect PATIENT PRIVACY. Data are available from Yuki Tanisaka, tanisaka1205@gmail.com for researchers who meet the criteria for access to confidential data.
